# Author Correction: Lipocalin 2 regulates mitochondrial phospholipidome remodeling, dynamics, and function in brown adipose tissue in male mice

**DOI:** 10.1038/s41467-023-44147-5

**Published:** 2023-12-12

**Authors:** Hongming Su, Hong Guo, Xiaoxue Qiu, Te-Yueh Lin, Chao Qin, Gail Celio, Peter Yong, Mark Senders, Xianlin Han, David A. Bernlohr, Xiaoli Chen

**Affiliations:** 1 Department of Food Science and Nutrition, University of Minnesota-Twin Cities, St. Paul, MN 55108 USA; 2 Barshop Institute for Longevity and Aging Studies, Department of Medicine, University of Texas Health Science Center at San Antonio, San Antonio, TX 78229-3900 USA; 3 University Imaging Centers, University of Minnesota-Twin Cities, Minneapolis, MN 55455 USA; 4 Department of Biochemistry, Molecular Biology and Biophysics, University of Minnesota-Twin Cities, Minneapolis, MN 55455 USA

**Keywords:** Energy metabolism, Fat metabolism, Mitochondria

Correction to: *Nature Communications* 10.1038/s41467-023-42473-2, published online 23 October 2023

The original version of the Supplementary Information associated with this Article contained errors in Supplementary Fig. 10. An earlier draft of this figure was mistakenly included rather than a revised version that had been updated during the peer review process.

The correct version of Supplementary Fig. 10 appears below:
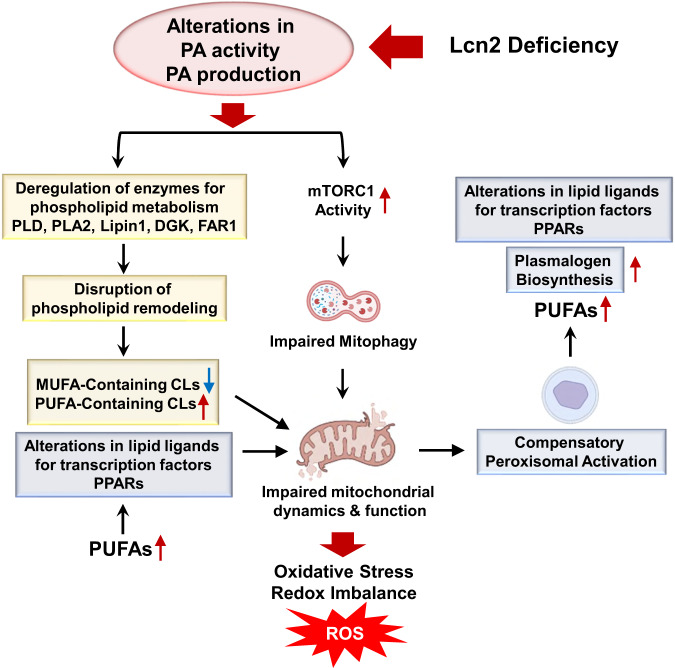


which replaces the previous incorrect version, which appears below:
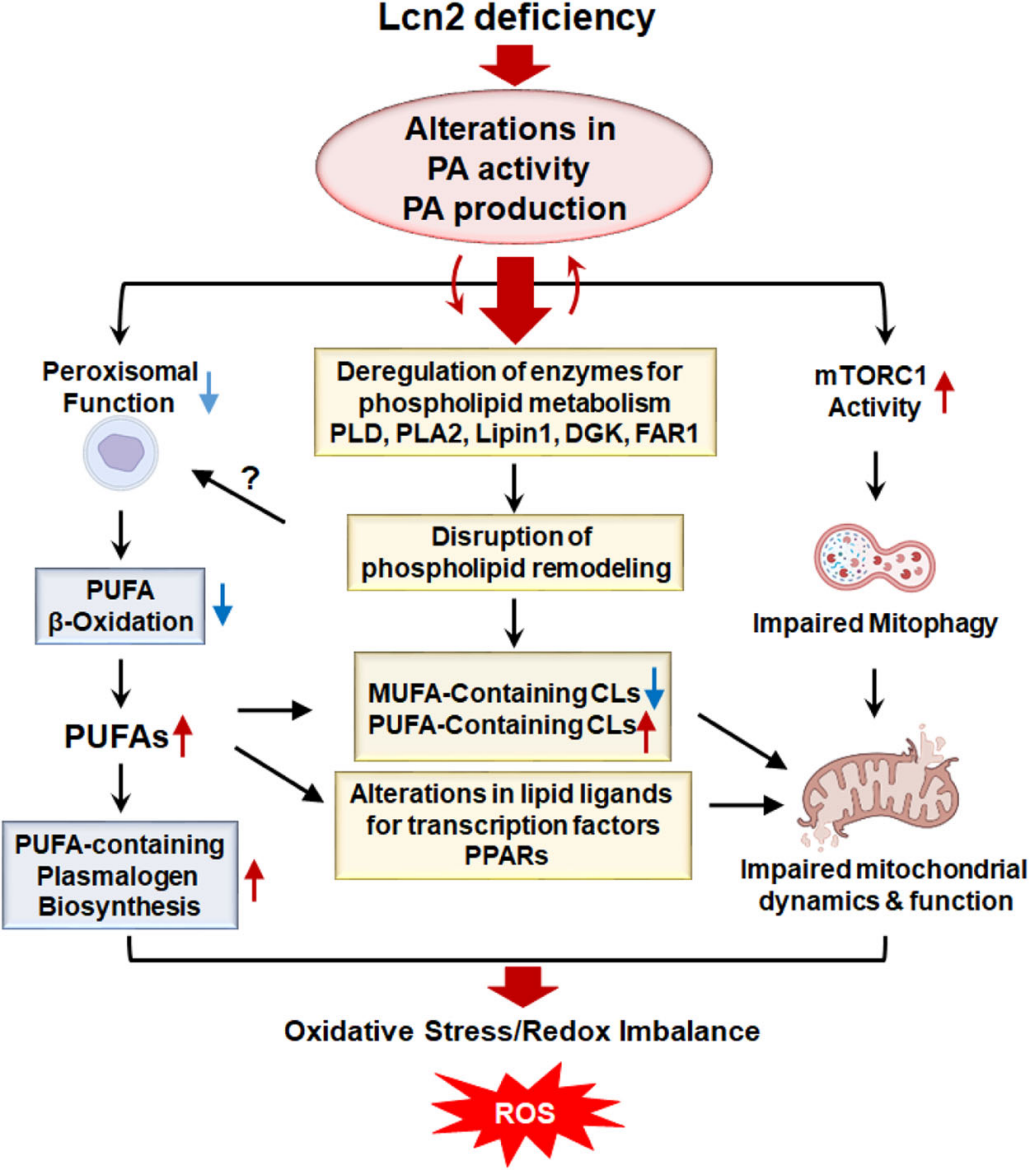


The HTML has been updated to include a corrected version of the Supplementary Information.

### Supplementary information

Supplementary Information

