# Supplementary material for: Lipocalin 2 regulates mitochondrial phospholipidome remodeling, dynamics, and function in brown adipose tissue in male mice
Source: Nat Commun. 2023 Oct 23;14:6729. doi: 10.1038/s41467-023-42473-2 (PMC10593768; doi:10.1038/s41467-023-42473-2)
Supplement: Supplementary file 1 — Supplementary Information [file 41467_2023_42473_MOESM1_ESM.pdf]

Supplementary Figure 1

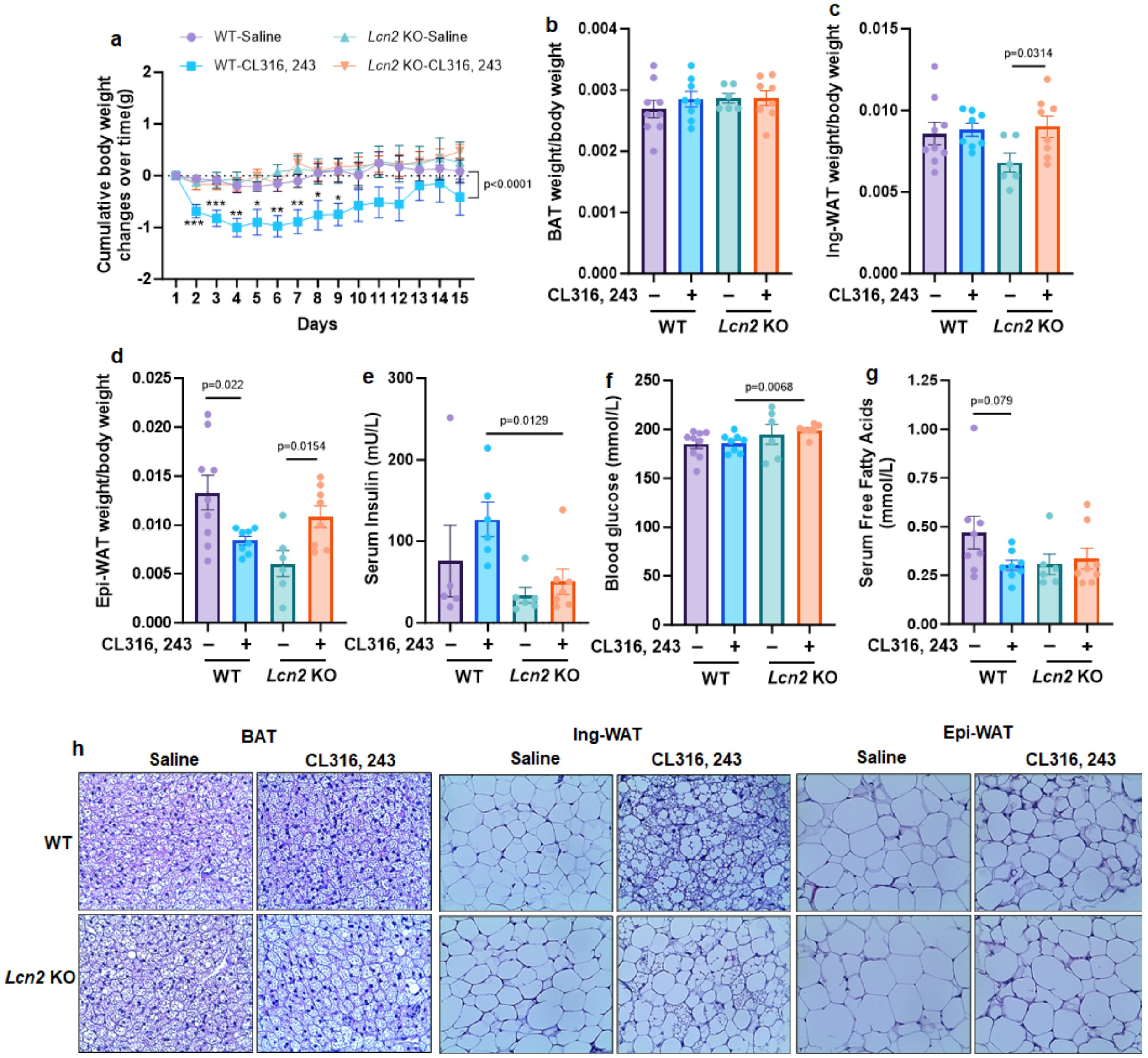

Supplementary Figure 1. Metabolic effect of CL316, 243 in *Lcn2* KO mice. Body weight changes during 14 days of CL316, 243 (0.5mg/kg BW) via i.p. injection once a day (a). Body fat mass, serum insulin, glucose, and fatty acid levels in WT and *Lcn2* KO mice after 14 days of CL316, 243 treatment (b-g). Representative H&E staining of BAT, Ing-WAT, and Epi-WAT of mice after 14 days of CL316, 243 treatment (h). Results are presented as mean ± SEM. Statistical significance was assessed by one-way ANOVA with Tukey's multiple comparison test for multiple groups for subfigure a. Student's t test was performed to test differences between two independent groups. All tests were two-sided. n=9 (saline-treated WT mice), n=8 (CL316, 243-treated WT mice), n=6 (saline-treated *Lcn2* KO mice), n=8 (CL316, 243-treated *Lcn2* KO mice). Samples used for all the measurements indicated in subfigures a-h are from the same set of mice. Source data are provided as a Source data file.

Supplementary Figure 2

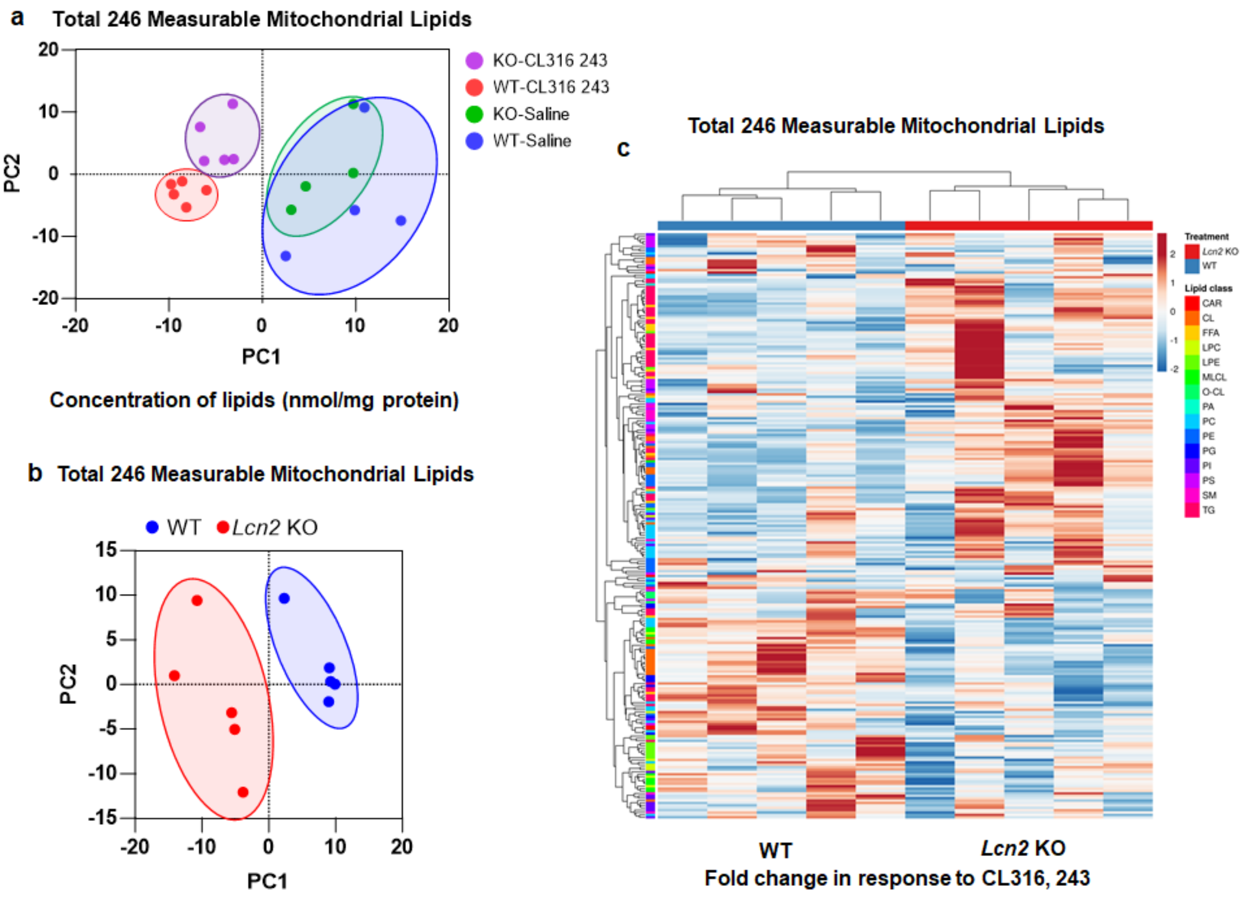

Supplementary Figure 2. Global analysis of alterations of total measurable mitochondrial lipids in BAT of *Lcn2* KO mice. PCA on the concentration of total 246 measurable lipids (a) in BAT. PCA (b) and hierarchical clustering (c) on fold changes of total 246 measurable lipids in response to CL316,243 in BAT of mice treated with or without CL316,243 for 14 days. n=4 (saline-treated WT and *Lcn2* KO mice), n=5 (CL316, 243-treated WT and *Lcn2* KO mice). Source data are provided as a Source data file.

Supplementary Figure 3

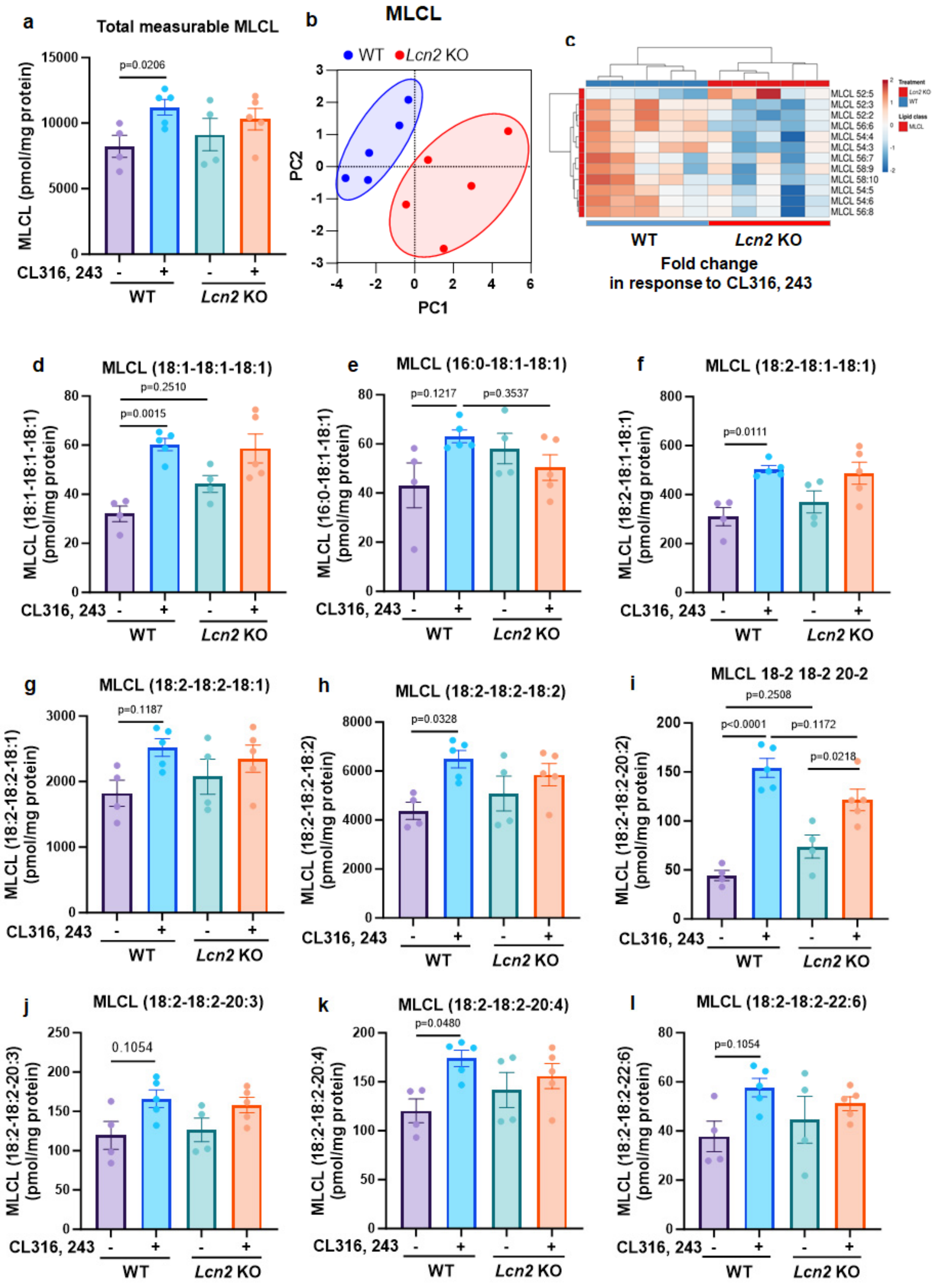

Supplementary Figure 3. **Effect of *Lcn2* deficiency on CL316, 243-induced changes in monolysocardiolipin (MLCL) in BAT.** Global analysis of total MLCL species (a), PCA (b), and hierarchical clustering (c). CL316, 243-induced changes in the content of individual MLCL (d-l). Results are presented as mean ± SEM. Student's t test was performed to test differences between two independent groups. All tests were two-sided. n=4 (saline-treated WT and *Lcn2* KO mice), n=5 (CL316, 243-treated WT and *Lcn2* KO mice). Source data are provided as a Source data file.

Supplementary Figure 4

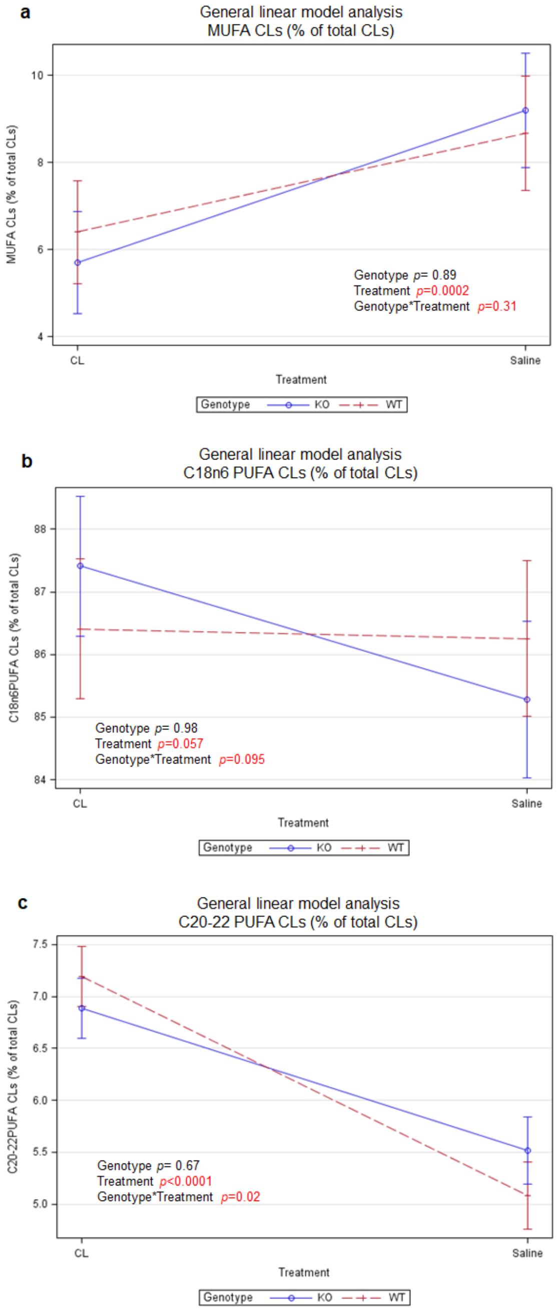

Supplementary Figure 4. General linear models analysis of cardiolipins with different categories of fatty acids. MUFA-CL (A), C18n6 PUFA-CL (B), and C20-22 PUFA-CL (C) percentage of total CLs. General linear models analysis was conducted to determine genotype and treatment interaction. Source data are provided as a Source data file.

Supplementary Figure 5

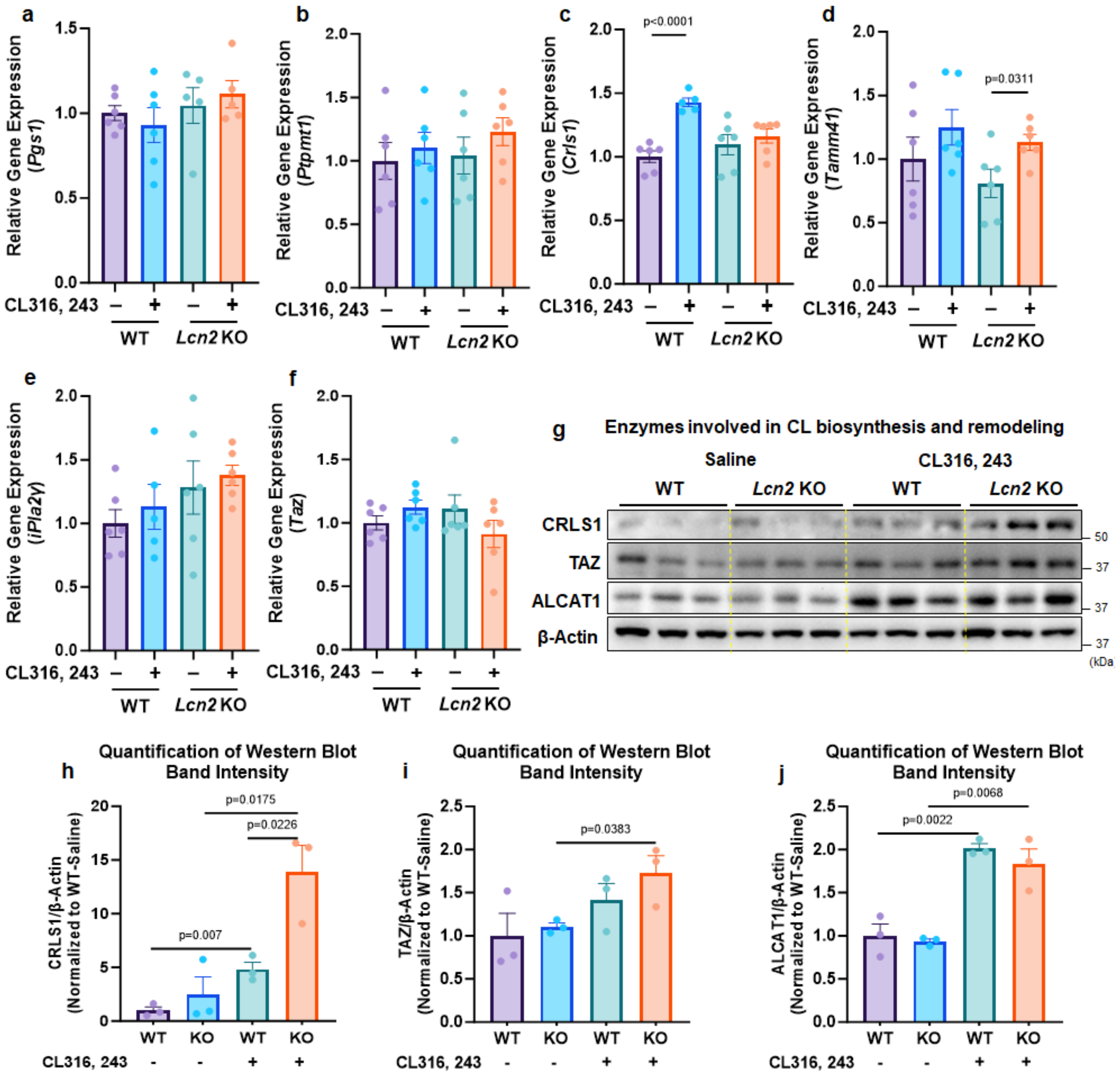

Supplementary Figure 5. Effect of *Lcn2* deficiency on the expression of genes and proteins involved in CL biosynthesis and remodeling in BAT. Expression of genes (a-f) and enzymes (g) involved in CL biosynthesis and remodeling in BAT of mice treated with or without CL316,243 for 14 days. Quantification of western-blotting band intensity (h-j) (n=3). Results are presented as mean ± SEM. Student's t test was performed to test differences between two independent groups. All tests were two-sided. n=6 for both saline- and CL316 243-treated WT and *Lcn2* KO mice. Source data are provided as a Source data file.

Supplementary Figure 6

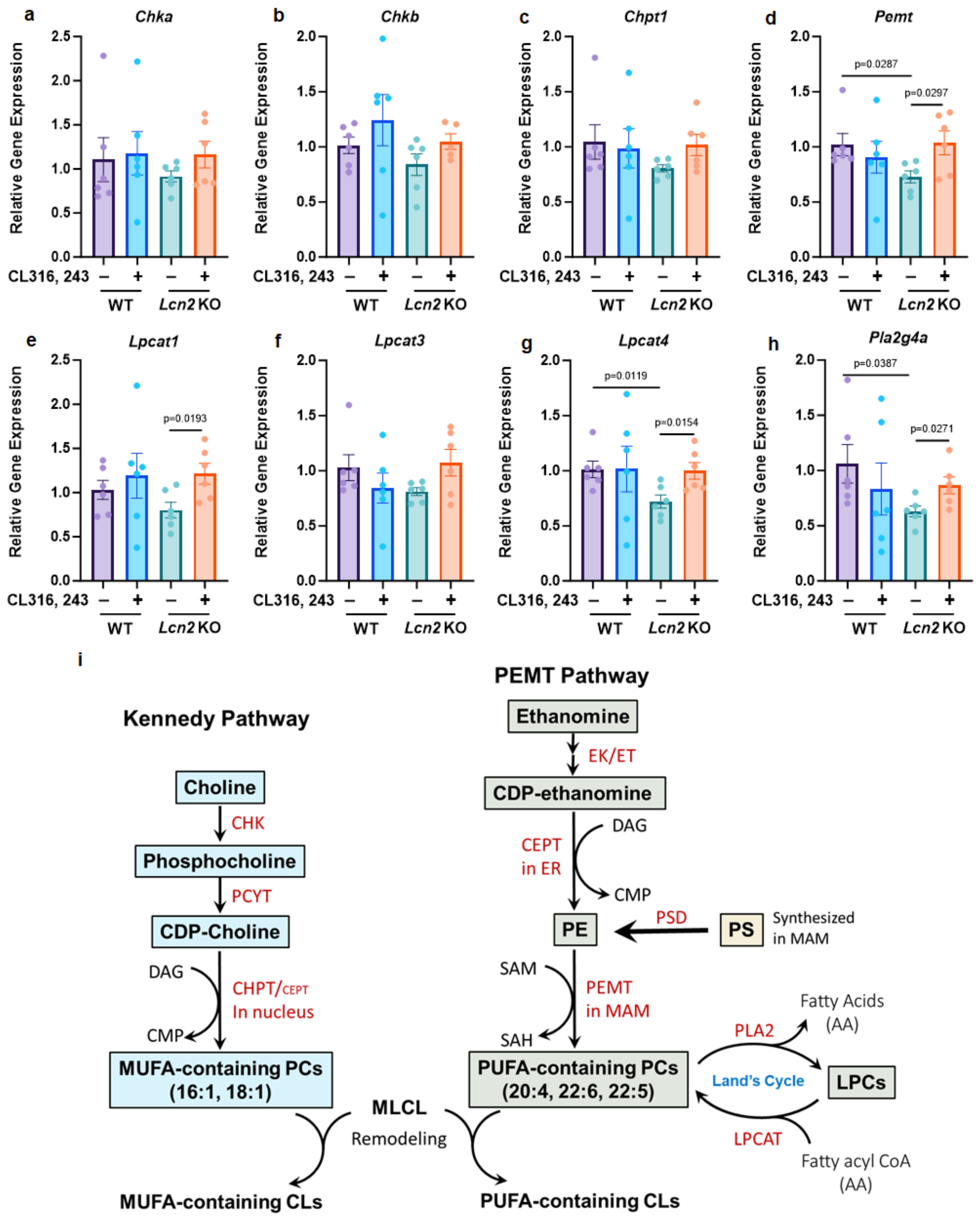

Supplementary Figure 6. Effect of *Lcn2* deficiency on Kennedy pathway and PEMT pathway of PC and PE biosynthesis and MLCL remodeling in BAT. (a-c) Gene expression of enzymes involved in PC biosynthesis in Kennedy pathway. (d) Gene expression of enzymes involved in PC biosynthesis in PEMT pathway. (e-h) Expression of genes in Land's cycle for PC-containing AA in BAT of mice treated with or without CL316, 243 for 14 days. Results are presented as mean ± SEM. Student's t test was performed to test differences between two independent groups. All tests were two-sided. n=6 for both saline- and CL316, 243-treated WT and *Lcn2* KO mice. (I) Schematic representation of biosynthetic pathways of PC and PE and MlCL remodeling. Source data are provided as a Source data file.

Supplementary Figure 7

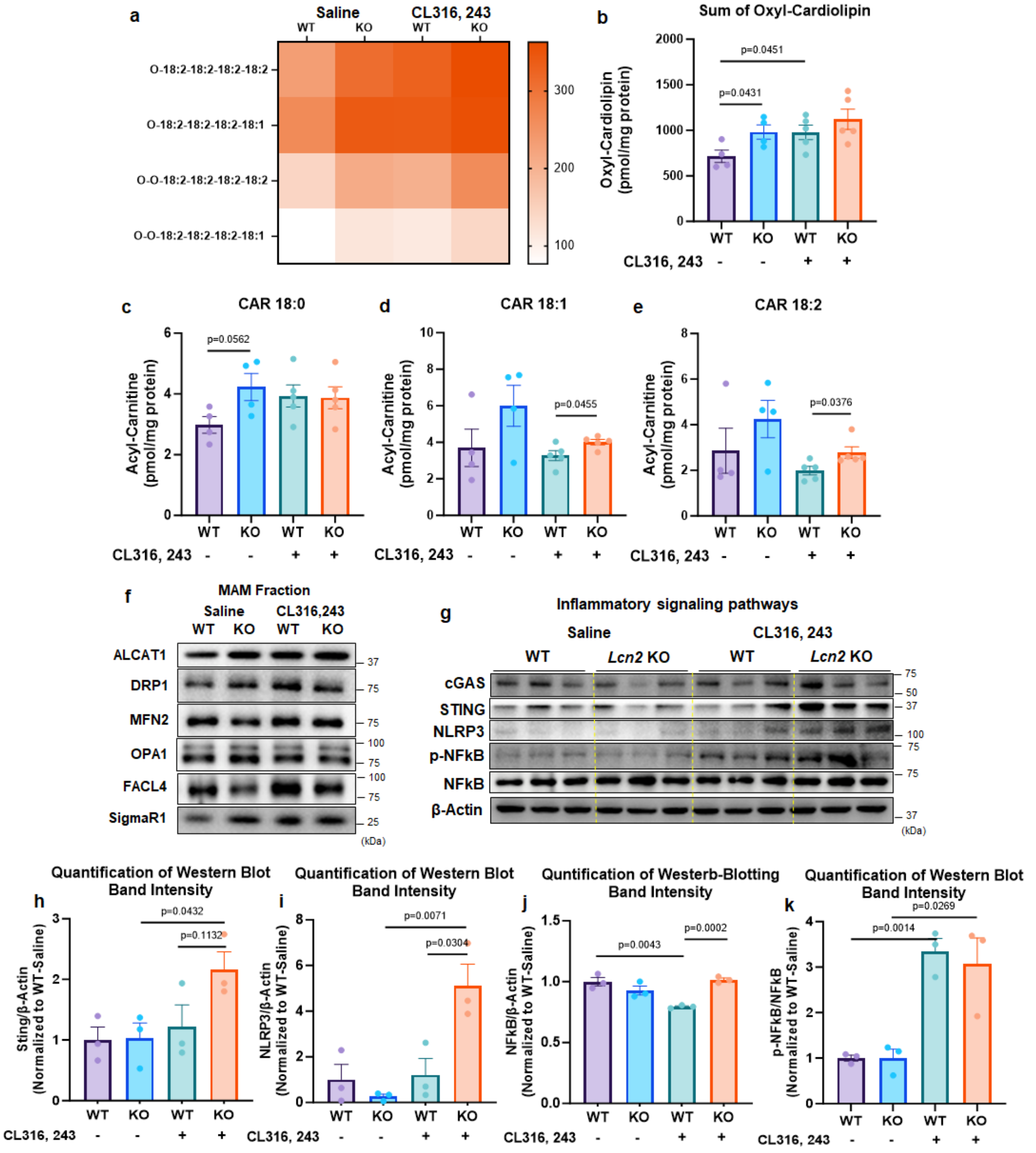

Supplementary Figure 7. Alterations of mitochondrial oxyl-cardiolipin and acyl-carnitine levels and inflammatory pathway activation in BAT. Individual (a) and total (b) oxyl-cardiolipin content. Acyl-carnitine levels in mitochondria isolated from BAT of WT and Lcn2 KO mice treated with Saline or CL316, 243 for 14 days (c-e). Proteins involved mitochondrial dynamics and MAM protein markers in MAM (f) isolated from BAT and inflammatory cGAS-STING and NFkB signaling pathway activation (g) in BAT of mice treated with and without CL316, 243 for 14 days. Quantification of western-blotting band intensity (h-k) (n=3). Results are presented as mean ± SEM. Student's t test was performed to test differences between two independent groups. All tests were two-sided. n=4 (saline-treated WT and *Lcn2* KO mice), n=5 (CL316, 243-treated WT and *Lcn2* KO mice). Source data are provided as a Source data file. CAR: Acyl-Carnitine

Supplementary Figure 8

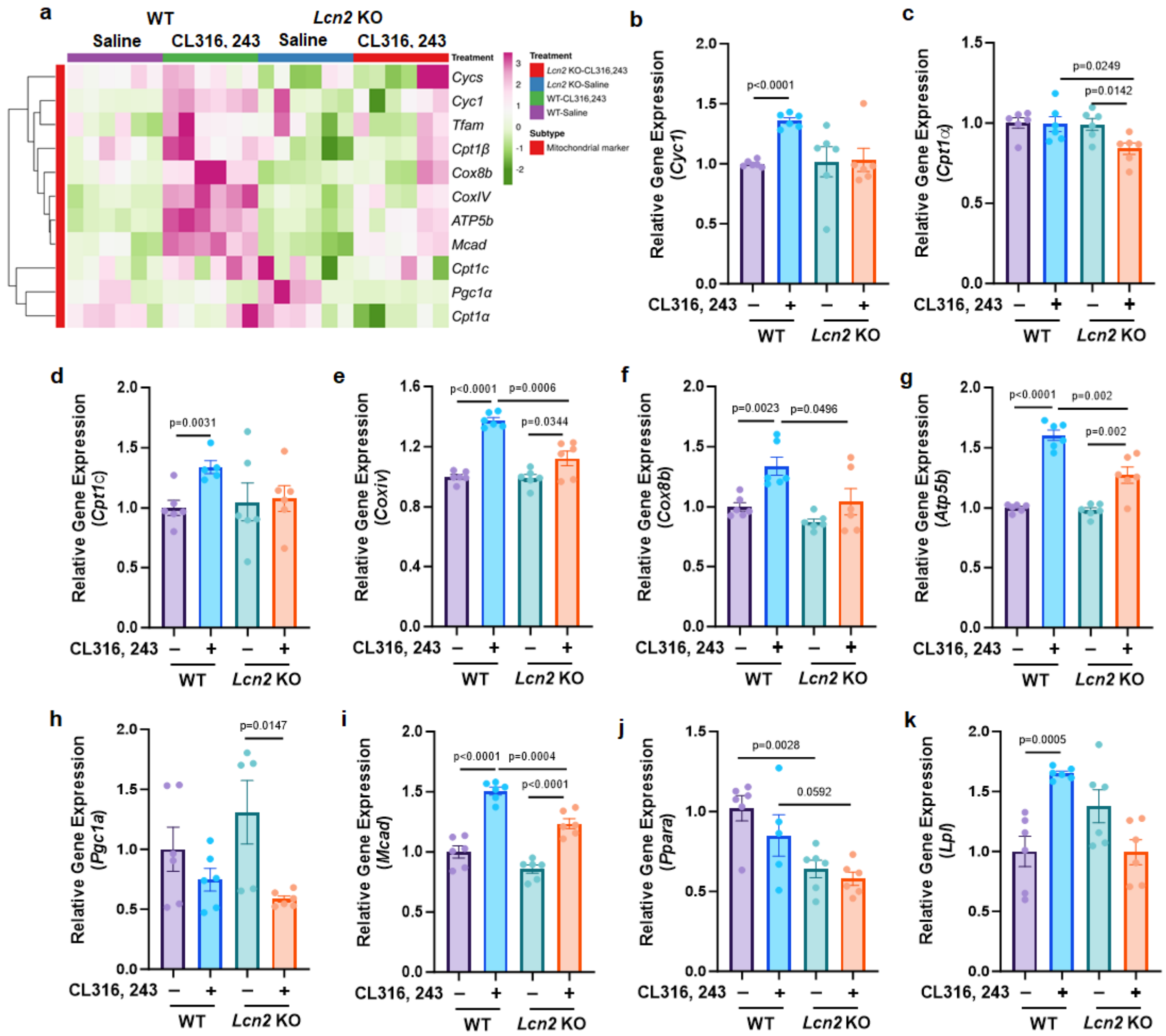

Supplementary Figure 8. Effect of *Lcn2* deficiency on the expression of genes involved in mitochondrial biogenesis and fatty acid oxidation in BAT. Hierarchical clustering of mitochondrial gene expression in BAT in response to CL316, 243 (a). Expression levels of individual genes involved in mitochondrial biogenesis and fatty acid oxidation in BAT of mice treated with or without CL316, 243 for 14 days (b-k). Results are presented as mean ± SEM. Student's t test was performed to test differences between two independent groups. All tests were two-sided. n=6 for both saline- and CL316, 243-treated WT and *Lcn2* KO mice. Source data are provided as a Source data file.

Supplementary Figure 9

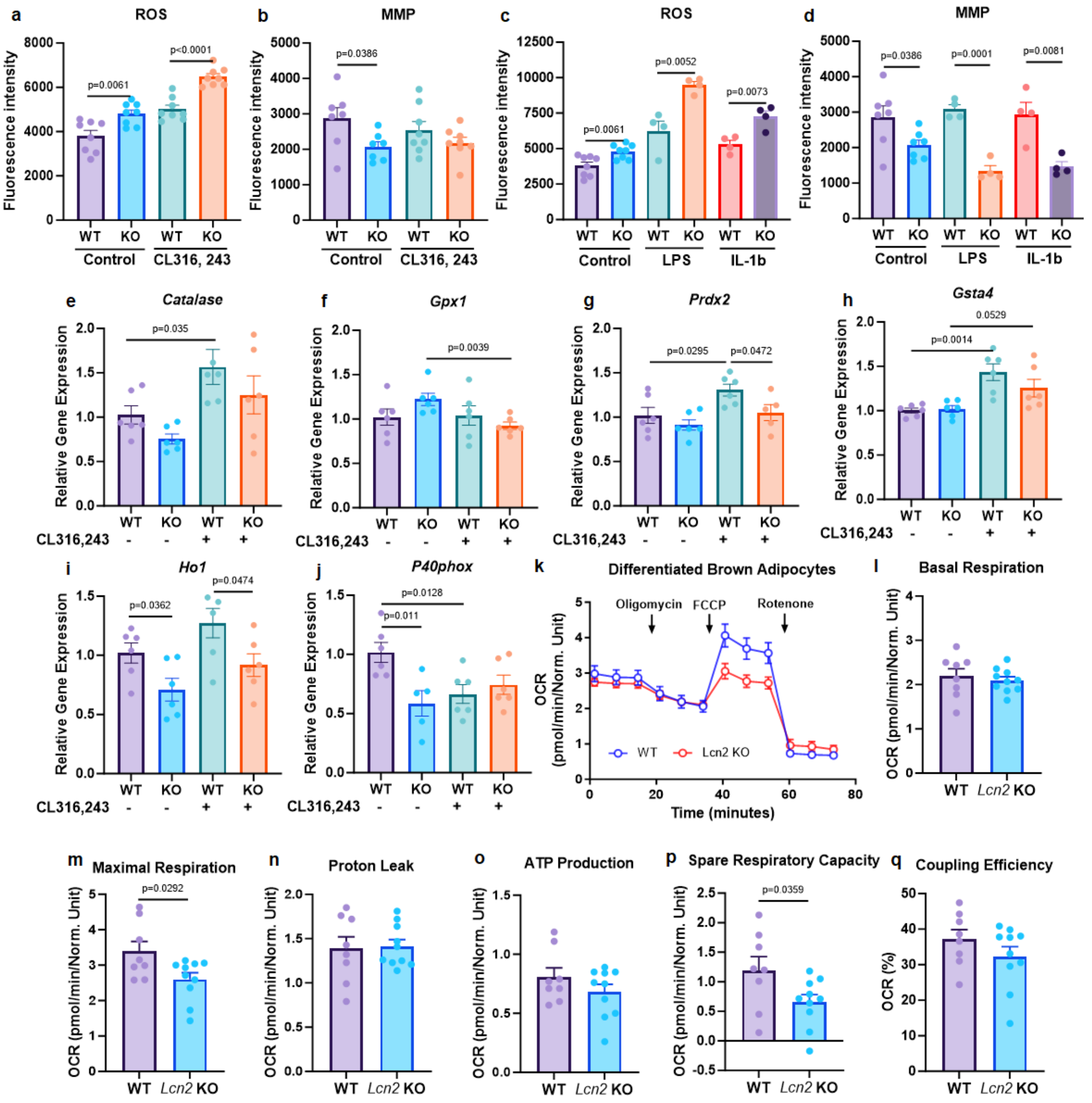

Supplementary Figure 9. Effect of *Lcn2* deficiency on oxidative stress, respiratory capacity, and anti-oxidant enzymes in differentiated brown adipocytes. The levels of reactive oxygen species (ROS) and mitochondrial membrane potential (MMP) in brown adipocytes treated with or without CL316, 243 (1 µM), LPS (1 µg/mL), or IL-1β (1 ng/mL) for 12h (a-d). The mRNA expression of anti-oxidant enzymes in BAT of mice treated with or without CL316, 243 for 14 days (n=6 for both saline- and CL316, 243-treated mice) (e-j). Seahorse analysis of respiratory capacity in primary brown adipocytes differenced from SV cells of WT and *Lcn2* KO mice (k-q). Brown adipocytes were treated with CL 316, 243 (1 µM) for 6 hours, followed by 1 µM oligomycin, 2 µM FCCP and 0.5 µM Rotenone/antimycin A treatment sequentially. The experiments were repeated twice and yielded similar results. Each experiment contained at least 4 replicates. Results are presented as mean ± SEM. Student's t test was performed to test differences between two independent groups. All tests were two-sided. Source data are provided as a Source data file.

Supplementary Figure 10

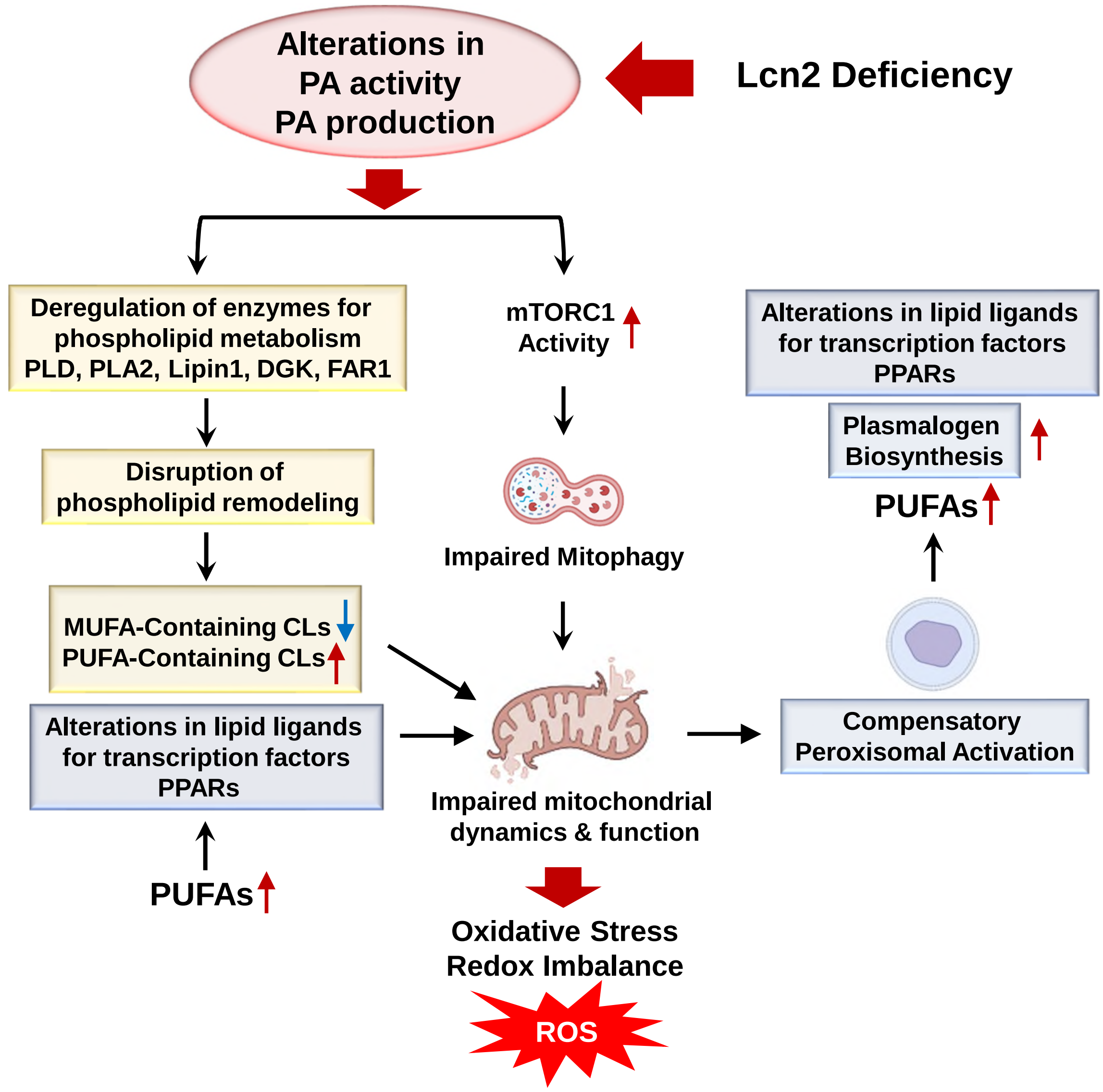

Supplementary Figure 10. A graphic summary of data interpretation for deregulated phospholipid metabolism and remodeling, mitochondrial dysfunction, and oxidative stress in Lcn2 KO BAT. The cartoons in this figure were created with BioRender.com.

Supplementary Table 1

Gene name	Forward primer	Reverse primer
Pgs1	GTTCCTCAGGTCACCTCCC	CCATCACAACTCGCCTCTTGG
Taz	CCCCCGCTTTGGACAGAAAAT	AGGCTGGAAATGATTGTGGAG
TAMM41	GAGCCACTACTCCTTCCTCAA	CCAGTGCTAATAACCCCGTACTT
PTPMT1	ACTATGAACGAGGAGTACGAGAC	GTTGGGACCCCAGTCATGTC
iPLA2 γ	CTCTATCGAAAGTTGGGCTCAGA	TCCCACGTGTTACTGTCATAAAAC
Crls1	GGGCTACCTGATTCTTGAAGA	GGCCCAGTTTCGAGCAATAA
CPT1α	CCTCCCTGGGCATGATTG	ACGCCACTCACGATGTTCTTC
CPT1c	AGAGAAGCCTGCCAGTTTGTGAGA	TGTACAGTGCAAAGAGGTGACGGT
COXIV	ATGTCACGATGCTGTCTGCC	GTGCCCCTGTTCATCTCGGC
COX8b	AGGAGTGCGACCCCGAGAATC	GGCTAAGACCCATCCTGCTGG
ATP5b	GCAAGGCAGGGACAGCAGA	CCCAAGGTCTCAGGACCAACA
PGC-1a	ACCGTAAATCTGCGGGATGATGGA	AGTCAGTTTCGTTCGACCTGCGTA
MCAD	AAGCCACGAAGTATGCCCTG	CCATAGCCTCCGAAAATCTG
CYC1	ATTTCAACCCTTACTTTCCCG	CCACTTATGCCGCTTCATGGC
LPL	TGAGAAAGGGCTCTGCCTGA	GGGCATCTGAGAGCGAGTCTT
β-actin	AACCGTGAAAAGATGACCCAGAT	CACAGCCTGGATGGCTACGTA
Lpcat1	GGCTCCTGTTCGCTGCTTT	TTCACAGCTACACGGTGGAAG
Lpcat3	CTACCCGTTGGCTCTGTTTTAC	TGAAGCACGACACATAGCAAG
Lpcat4	CTCATCCGATACCCCAACAGT	GGGAGGAACTCTACATCCACG
Chka	AGCCTCGGAAAGTGCTCTTG	GTCGGCCTTGGGGAAAGATG
Chkb	AGGATGCTAAGTGCCCAGAG	TCACGGGACAAACGCTCAG
Chpt1	ACTGAGATCCAGGTAGCTTTAGT	GTAGACCCATTCTTGCCAACA
PEMT	TGGCTGCTGGGTTACATGG	GCTTCCGAGTTCTCTGCTCC
Pla2g4a	CAGCACATTATAGTGGAACACCA	AGTGTCCAGCATATCGCCAAA

DGKα	GATGAACAGATTTTGCCAGGGA	GTAGCAGTACACATCACTGAGAC
DGKγ	ATGAGCGAAGAACAATGGGTC	GGGCTTGTGTGGGTCATACTG
DGKζ	CTCTTTGGGCACAGGAAAGC	TGCTGACTCACTCCAGTCCA
DGKδ	CCAGCCACCTGGGTACATTC	CAGGGTGCAGGGAAAAGGAG
DGKε	TGGTCCTATGGACGCTGTG	CTGAACAGGTCGGTGTCACG
DGKη	CACCTTCTGTAACGTGTGCAG	CCATCCTCGTCCTCTATGATGTC
p40 phox	GCCGCTATCGCCAGTTCTAC	GCAGGCTCAGGAGGTTCTTC
Gsta4	CTGTACTGTCCGACTTCCCTC	CTCTGACTTCCGGGTTGCAG
Prdx2	CGTTGCTTACAGGGGTCTCTTTATC	CATTGGGCTTGATGGTGTCACT
Catalase	CCGACCAGGGCATCAAAA	GAGGCCATAATCCGGATCTTC

Supplementary Table 2

Antibodies	Source	Identifier
Rabbit DRP1 (D8H5) monoclonal Antibody, WB (1:1000)	Cell Signaling Technology	Catalog #5391, Lot: 1
Rabbit OPA1 (D6U6N) monoclonal Antibody, WB (1:1000)	Cell Signaling Technology	Catalog #80471, Lot:1
Rabbit Mitofusin-2 (D2D10) monoclonal Antibody, WB (1:1000)	Cell Signaling Technology	Catalog #9482, Lot:3
Goat Polyclonal Lipocalin-2/NGAL Antibody, WB (1:800)	R&D Systems	Catalog #AF1857, Lot: JZP0622061
Rabbit polyclonal β-actin Antibody, WB (1:1000)	Cell Signaling Technology	Catalog #4967, Lot: 20
Rabbit Polyclonal FACL4 Antibody, WB (1:1000)	Novus Biologicals	Catalog #NBP2-16401, Lot: 39568
Monoclonal Phospho-DRP1 (Ser616) (D9A1) Rabbit mAb	Cell Signaling Technology	Catalog #4494, Lot: 4
Monoclonal SigmaR1 (D4J2E) Rabbit mAb	Cell Signaling Technology	Catalog #61994, Lot:1
Rabbit Monoclonal cGAS (D3O8O) Antibody, WB (1:1000)	Cell Signaling Technology	Catalog #31659, Lot: 2
Rabbit Monoclonal Sting (D1V5L) Antibody, WB (1:1000)	Cell Signaling Technology	Catalog #50494, Lot:1
Rabbit Monoclonal NLRP3 (D4D8T) Antibody, WB (1:1000)	Cell Signaling Technology	Catalog #15101, Lot: 3
Rabbit Monoclonal p-NFkB p65 (Ser536) (93H1) Antibody, WB (1:1000)	Cell Signaling Technology	Catalog #3033, Lot:19
Mouse Monoclonal NFkB (D14E12) Antibody, WB (1:1000)	Cell Signaling Technology	Catalog #8242, Lot: 16
Mouse Monoclonal Mito-PLD (26C46-6) Antibody, WB (1:1000)	MBL	Catalog #M207-3, Lot: 001
Rabbit polyclonal Lipin1 Antibody, WB (1:1000)	Cell Signaling Technology	Catalog #5195

Rabbit polyclonal p-PLA2G4A Antibody, WB (1:1000)	AB colonal	Catalog #AP0968, Lot: 3516901206
Rabbit polyclonal PLA2G4A Antibody, WB (1:1000)	AB colonal	Catalog #A0394, Lot: 0081390101
Rabbit Monoclonal COX2 (D5H5) Antibody, WB (1:1000)	Cell Signaling Technology	Catalog #12282, Lot: 6
Rabbit Monoclonal p70 S6k Antibody, WB (1:1000)	Cell Signaling Technology	Catalog #9202, Lot: 15
Rabbit Monoclonal p-p70 S6k Antibody, WB (1:1000)	Cell Signaling Technology	Catalog #9204, Lot: 26
Rabbit Monoclonal p-ERK (197G2) Antibody, WB (1:1000)	Cell Signaling Technology	Catalog #4377, Lot: 10
Rabbit Monoclonal ERK (137F5) Antibody, WB (1:1000)	Cell Signaling Technology	Catalog #4695, Lot: 14
Rabbit Monoclonal LC3 (D3U4C) Antibody, WB (1:1000)	Cell Signaling Technology	Catalog #12741, Lot: 3
Rabbit polyclonal CRLS1 Antibody, WB (1:1000)	Proteintech	Catalog # 51055-1-AP, Lot: 00001490
Mouse Monoclonal Taz (1F1) Antibody, WB (1:1000)	Santa Cruz	Catalog #sc-293183, Lot: J0121
Rabbit polyclonal FAR1 Antibody, WB (1:1000)	AB colonal	Catalog #A16284, Lot: 5500029476
Rabbit polyclonal α/β-Tubulin Antibody, WB (1:1000)	Cell Signaling Technology	Catalog #2148, Lot: 8
Rabbit polyclonal Phospho-mTOR (Ser2481) Antibody, WB (1:1000)	Cell Signaling Technology	Catalog #2974, Lot: 13
Rabbit monoclonal mTOR (7C10) Antibody, WB (1:1000)	Cell Signaling Technology	Catalog #2983, Lot: 19
Rabbit monoclonal Raptor (24C12) Antibody, WB (1:1000)	Cell Signaling Technology	Catalog #2280, Lot: 13
Rabbit monoclonal Rictor (53A2) Antibody, WB (1:1000)	Cell Signaling Technology	Catalog #2114, Lot: 7

Rabbit monoclonal GβL (86B8) Antibody, WB (1:1000)	Cell Signaling Technology	Catalog #3274, Lot: 4
Rabbit monoclonal Phospho-mTOR (D9C2) (Ser2448) Antibody, WB (1:1000)	Cell Signaling Technology	Catalog #5536, Lot: 12
Rabbit polyclonal Calnexin Antibody, IF (1:200)	Abcam	Catalog# ab75801, Lot: GR280338-30
Rabbit polyclonal Tom20 (FL-145) Antibody, IF (1:50)	Santa Cruz	Catalog #sc-11415, Lot: A0915
Rat polyclonal ALCAT1 Antibody, WB (1:1000)	Gifted by Dr. Yuguang Shi	Gifted by Dr. Yuguang Shi
Anti-rabbit HRP secondary Antibody, WB (1:10000)	R&D Systems	Catalog#HAF008, Lot: FIN1922041
Anti-mouse HRP secondary Antibody, WB (1:10000)	R&D Systems	Catalog#HAF007, Lot: FIM3222041
Anti-rat HRP secondary Antibody, WB (1:10000)	R&D Systems	Catalog#HAF005, Lot: XGO1521031
Anti-goat HRP secondary Antibody, WB (1:10000)	R&D Systems	Catalog#HAF019, Lot: 102M4823
