# Supplementary material for: Lipocalin 2 regulates mitochondrial phospholipidome remodeling, dynamics, and function in brown adipose tissue in male mice
Source: Nat Commun. 2023 Oct 23;14:6729. doi: 10.1038/s41467-023-42473-2 (PMC10593768; doi:10.1038/s41467-023-42473-2)
Supplement: Supplementary file 3 — Description of Additional Supplementary Files [file 41467_2023_42473_MOESM3_ESM.pdf]

Description of Additional Supplementary Files

File Name: Supplementary Data 1

Description: The lipidomics data for measurable lipid species in this study, including PC, LPC, PE, LPE, SM, CAR, CL, Lyso CL, O-CL, PA, PG, PI, PS, TAG and FA. Column A lists the identified lipids within each lipid class. Column B displays the measured value of m/z for each lipid. Column C represents the determined molecular weight for each lipid. Column D to U represent the levels of lipid species for each sample.

File Name: Supplementary Data 2

Description: The lipidomics data for measureable lipid species of diacylglycerol and phosphatidic acid. Column A lists the identified lipid species in each lipid class. Column B displays the measured value of m/z for each lipid. Column C represents the determined molecular weight of each lipid. Column D to W represent the levels of lipid species for each sample.
