# Supplementary material for: Lipocalin 2 regulates mitochondrial phospholipidome remodeling, dynamics, and function in brown adipose tissue in male mice
Source: Nat Commun. 2023 Oct 23;14:6729. doi: 10.1038/s41467-023-42473-2 (PMC10593768; doi:10.1038/s41467-023-42473-2)
Supplement: Supplementary file 6 — Reporting Summary [file 41467_2023_42473_MOESM6_ESM.pdf]

nature portfolio

Corresponding author(s): Xiaoli Chen

Last updated by author(s): Sep 14, 2023

Reporting Summary

Nature Portfolio wishes to improve the reproducibility of the work that we publish. This form provides structure for consistency and transparency in reporting. For further information on Nature Portfolio policies, see our Editorial Policies and the Editorial Policy Checklist.

Statistics

For all statistical analyses, confirm that the following items are present in the figure legend, table legend, main text, or Methods section.

n/a	Confirmed	
☐	☒	The exact sample size (*n*) for each experimental group/condition, given as a discrete number and unit of measurement
☐	☒	A statement on whether measurements were taken from distinct samples or whether the same sample was measured repeatedly
☐	☒	The statistical test(s) used AND whether they are one- or two-sided<br>*Only common tests should be described solely by name; describe more complex techniques in the Methods section.*
☐	☒	A description of all covariates tested
☐	☒	A description of any assumptions or corrections, such as tests of normality and adjustment for multiple comparisons
☐	☒	A full description of the statistical parameters including central tendency (e.g. means) or other basic estimates (e.g. regression coefficient) AND variation (e.g. standard deviation) or associated estimates of uncertainty (e.g. confidence intervals)
☐	☒	For null hypothesis testing, the test statistic (e.g. F, t, r) with confidence intervals, effect sizes, degrees of freedom and P value noted<br>*Give P values as exact values whenever suitable.*
☒	☐	For Bayesian analysis, information on the choice of priors and Markov chain Monte Carlo settings
☐	☒	For hierarchical and complex designs, identification of the appropriate level for tests and full reporting of outcomes
☐	☒	Estimates of effect sizes (e.g. Cohen's *d*, Pearson's *r*), indicating how they were calculated

Our web collection on statistics for biologists contains articles on many of the points above.

Software and code

Policy information about availability of computer code

Data collection	Western blots were visualized by the iBright 750 imaging system (Thermo Fisher Scientific) or by the SRX-101A film processor (Konica Minolta). Quantitative PCR was performed using QuantStudio 3 Real-time PCR system (Applied Biosystems, Thermo Fisher Scientific). Biochemical colorimetric assays were detected by Synergy HT 96-well microtiter plate reader (Bio-Tek). Fluorescence intensity was detected by Tecan infinite 200Pro microplate reader (Tecan). Cellular respiration was determined by XF96 Extracellular Flux Analyzer (Agilent Seahorse). Tissue sections were visualized by Leica EC3 microscope (Leica Microsystems). Transmission electron microscopy (TEM) was performed using JEOL JEM-1400plus electron microscope (JEOL). The mass spectrometric analysis was performed on a triple quadrupole mass spectrometer (TSQ Altis, Thermo Fisher Scientific) and a hybrid quadrupole-Orbitrap mass spectrometer (Q-Exactive, Thermo Fisher). Confocal microscopy was performed with Nikon A1 confocal microscope (Nikon).
Data analysis	Statistical analysis was performed by GraphPad Prism 10 or SAS software v9.4. Heatmaps were plotted using Clustvis. Western blots and mitochondrial size were quantified by ImageJ v 1.53t. Real-time PCR was analyzed by QuantStudio Design & Analysis Software v1.5.1 (Thermo Fisher Scientific). Calorimetric assays were analyzed by Gen5 2.03. Fluorescence intensity was acquired by Tecan i-control. Cell respiratory was analyzed by Wave 2.6.3 (Agilent Seahorse). Confocal images were acquired by NIS Elements (Nikon). TEM images were acquired by AMT Capture Engine software.

For manuscripts utilizing custom algorithms or software that are central to the research but not yet described in published literature, software must be made available to editors and reviewers. We strongly encourage code deposition in a community repository (e.g. GitHub). See the Nature Portfolio guidelines for submitting code & software for further information.

Data

Policy information about availability of data

All manuscripts must include a data availability statement. This statement should provide the following information, where applicable:

- Accession codes, unique identifiers, or web links for publicly available datasets
- A description of any restrictions on data availability
- For clinical datasets or third party data, please ensure that the statement adheres to our policy

Data are available within the Article, Supplementary information, or Supplementary data. The datasets generated and/or analyzed during the current study, which have not been deposited in a public repository can be obtained from the corresponding author upon reasonable request. Source data are included with this paper.

Research involving human participants, their data, or biological material

Policy information about studies with human participants or human data. See also policy information about sex, gender (identity/presentation), and sexual orientation and race, ethnicity and racism.

Reporting on sex and gender	N/A
Reporting on race, ethnicity, or other socially relevant groupings	N/A
Population characteristics	N/A
Recruitment	N/A
Ethics oversight	N/A

Note that full information on the approval of the study protocol must also be provided in the manuscript.

Field-specific reporting

Please select the one below that is the best fit for your research. If you are not sure, read the appropriate sections before making your selection.

☒ Life sciences ☐ Behavioural & social sciences ☐ Ecological, evolutionary & environmental sciences

For a reference copy of the document with all sections, see nature.com/documents/nr-reporting-summary-flat.pdf

Life sciences study design

All studies must disclose on these points even when the disclosure is negative.

Sample size	Sample sizes were included in Figure legends. Sample sizes were calculated based on the effect size (the difference between 2 groups) and the significance level from our previous studies as well as similar studies reported in the literature (PMID: 30928474, 27008859, 20332347).
Data exclusions	No data were excluded from analyses.
Replication	Animal and cell culture experiments were independently repeated at least two to three times as indicated in figure legends to ensure reproducibility.
Randomization	Mice were allocated randomly into experimental groups. For the cell culture experiments, cell culture wells were randomly assigned to treatments in each independent round of experiment.
Blinding	In this study blinding was not possible because the treatment of animals/cells as well as data collection were frequently done by the same investigators. Lipidomics analyses were done by collaborators and they were unaware of the study design.

Reporting for specific materials, systems and methods

We require information from authors about some types of materials, experimental systems and methods used in many studies. Here, indicate whether each material, system or method listed is relevant to your study. If you are not sure if a list item applies to your research, read the appropriate section before selecting a response.

Materials & experimental systems

n/a	Involved in the study
☐	☒ Antibodies
☐	☒ Eukaryotic cell lines
☒	☐ Palaeontology and archaeology
☐	☒ Animals and other organisms
☒	☐ Clinical data
☒	☐ Dual use research of concern
☒	☐ Plants

Methods

n/a	Involved in the study
☒	☐ ChIP-seq
☒	☐ Flow cytometry
☒	☐ MRI-based neuroimaging

Antibodies

Antibodies used

Rabbit DRP1 (D8H5) monoclonal Antibody, WB (1:1000), Cell Signaling Technology, Catalog #5391, Lot: 1
Rabbit OPA1 (D6U6N) monoclonal Antibody, WB (1:1000), Cell Signaling Technology, Catalog #80471, Lot:1
Rabbit Mitofusin-2 (D2D10) monoclonal Antibody, WB (1:1000), Cell Signaling Technology, Catalog #9482, Lot:3
Goat polyclonal Lipocalin-2/NGAL Antibody, WB (1:800), R&D Systems, Catalog #AF1857, Lot: JZP0622061
Rabbit polyclonal β-actin Antibody, WB (1:1000), Cell Signaling Technology, Catalog #4967, Lot: 20
Rabbit Polyclonal FACL4 Antibody, WB (1:1000), Novus Biologicals, Catalog #NBP2-16401, Lot: 39568
Monoclonal Phospho-DRP1 (Ser616) (D9A1) Rabbit mAb, WB (1:1000), Cell Signaling Technology, Catalog #4494, Lot: 4
Monoclonal SigmaR1 (D4J2E) Rabbit mAb, WB (1:1000), Cell Signaling Technology, Catalog #61994, Lot:1
Rabbit monoclonal cGAS (D3O8O) Antibody, WB (1:1000), Cell Signaling Technology, Catalog #31659, Lot: 2
Rabbit monoclonal Sting (D1V5L) Antibody, WB (1:1000), Cell Signaling Technology, Catalog #50494, Lot:1
Rabbit monoclonal NLRP3 (D4D8T) Antibody, WB (1:1000), Cell Signaling Technology, Catalog #15101, Lot: 3
Rabbit monoclonal p-NFkB p65 (Ser536) (93H1) Antibody, WB (1:1000), Cell Signaling Technology, Catalog #3033, Lot:19
Mouse monoclonal NFkB (D14E12) Antibody, WB (1:1000), Cell Signaling Technology, Catalog #8242, Lot: 16
Mouse monoclonal Mito-PLD (26C46-6) Antibody, WB (1:1000), MBL, Catalog #M207-3, Lot: 001
Rabbit polyclonal Lipin1 Antibody, WB (1:1000), Cell Signaling Technology, Catalog #5195, Lot: 2
Rabbit polyclonal p-PLA2G4A Antibody, WB (1:1000), AB colonal, Catalog #AP0968, Lot: 3516901206
Rabbit polyclonal PLA2G4A Antibody, WB (1:1000), AB colonal, Catalog #A0394, Lot: 0081390101
Rabbit monoclonal COX2 (D5H5) Antibody, WB (1:1000), Cell Signaling Technology, Catalog #12282, Lot: 6
Rabbit monoclonal p70 S6k Antibody, WB (1:1000), Cell Signaling Technology, Catalog #9202, Lot: 15
Rabbit monoclonal p-p70 S6k Antibody, WB (1:1000), Cell Signaling Technology, Catalog #9204, Lot: 26
Rabbit monoclonal p-ERK (197G2) Antibody, WB (1:1000), Cell Signaling Technology, Catalog #4377, Lot: 10
Rabbit monoclonal ERK (137F5) Antibody, WB (1:1000), Cell Signaling Technology, Catalog #4695, Lot: 14
Rabbit monoclonal LC3 (D3U4C) Antibody, WB (1:1000), Cell Signaling Technology, Catalog #12741, Lot: 3
Rabbit polyclonal CRLS1 Antibody, WB (1:1000), Proteintech, Catalog #51055-1-AP, , Lot: 00001490
Mouse monoclonal Taz (1F1) Antibody, WB (1:1000), Santa Cruz, Catalog #sc-293183, Lot: J0121
Rabbit polyclonal FAR1 Antibody, WB (1:1000), AB colonal, Catalog #A16284, Lot: 5500029476
Rabbit polyclonal α/β-Tubulin Antibody, WB (1:1000), Cell Signaling Technology, Catalog #2148, Lot: 8
Rabbit polyclonal Phospho-mTOR (Ser2481) Antibody, WB (1:1000), Cell Signaling Technology, Catalog #2974, Lot: 13
Rabbit monoclonal mTOR (7C10) Antibody, WB (1:1000), Cell Signaling Technology, Catalog #2983, Lot: 19
Rabbit monoclonal Raptor (24C12) Antibody, WB (1:1000), Cell Signaling Technology, Catalog #2280, Lot: 13
Rabbit monoclonal Rictor (53A2) Antibody, WB (1:1000), Cell Signaling Technology, Catalog #2114, Lot: 7
Rabbit monoclonal GbL (86B8) Antibody, WB (1:1000), Cell Signaling Technology, Catalog #3274, Lot: 4
Rabbit monoclonal Phospho-mTOR (Ser2448) (D9C2) Antibody, WB (1:1000), Cell Signaling Technology, Catalog #5536, Lot: 12
Rat polyclonal ALCAT1 Antibody, WB (1:1000), Gifted by Dr. Yuguang Shi (ALCAT1 antibody was originally produced from rat strain by Dr. Hiroyuki Arai's lab, University of Tokyo, Japan)
Anti-rabbit HRP secondary Antibody, WB (1:10000), R&D Systems, Catalog#HAF008, Lot: FIN1922041
Anti-mouse HRP secondary Antibody, WB (1:10000), R&D Systems, Catalog#HAF007, Lot: FIM3222041
Anti-rat HRP secondary Antibody, WB (1:10000), R&D Systems, Catalog#HAF005, Lot: XGO1521031
Anti-goat HRP secondary Antibody, WB (1:10000), R&D Systems, Catalog#HAF019, Lot: 102M4823

Validation

All the commercial antibodies used in this study have been tested/validated by the manufacturers. Antibodies purchased from Cell Signaling Technology including Anti-DRP1, Anti-OPA1, Anti-Mitofusin-2, Anti-β-Actin, Anti- Phospho-DRP1 (Ser616), Anti-SigmaR1, Anti-cGAS, Anti-STING, Anti-NLRP3, Anti-p-NFkB p65 (Ser536), Anti-NFkB, Anti-LIPIN1, Anti-COX2, Anti-p-ERK, Anti-ERK, Anti-α/β-Tubulin, Anti-Raptor, Anti-Rictor, and Anti-GbL antibodies were validated by the manufacturers and the application of these antibodies was published by other research groups (This information is provided at www.cellsignal.com). Anti-MitoPLD antibody purchased from MBL was validated by the manufacturer and the application of this antibody was published by other research groups (This information is available at https://www.mblbio.com/bio/g/dtl/A/index.html?pcd=M207-3#u-pub). Anti-TAZ antibody purchased from Santa Cruz was validated by the manufacturer and the application of this antibody was published by other research groups (This information is provided at https://www.scbt.com/p/taz-antibody-1f1?requestFrom=search). Anti-CRLS1 Antibody purchased from Proteintech was validated by the manufacturer and the application of this antibody was published by other research groups (The information is available at https://www.ptglab.com/products/CRLS1-Antibody-51055-1-AP.htm). Anti-FACL4 antibody purchased from Novus Biologicals was validated by the manufacturer and the application of this antibody was published by other research groups (This information is provided at https://www.novusbio.com/products/facl4-antibody_nbp2-16401#datasheet). Antibodies including Anti-p-PLA2G4A, Anti-PLA2G4A and Anti-FAR1 purchased from AB Colonal were validated by the manufacturer and the application of these antibodies was published by other research groups (The information is provided at https://abclonal.com). Additionally, anti-Lipocalin-2/ NGAL antibody was purchased from the R&D systems and validated by the manufacturer as well as whole body knockout mouse model of target protein (LCN2) in the laboratory. Anti-Phospho-mTOR (Ser2481), anti-mTOR, anti-Phospho-mTOR (Ser2448), anti-LC3, anti-p70 S6k and anti-p-p70 S6k antibodies were previously validated by pharmacological

inhibitors treatment (Rapamycin or Torin) in the laboratory. ALCAT1 antibody was kindly provided by Dr. Yuguang Shi by UT Health Science Center at San Antonio and validated by whole body knockout mouse model of target protein (ALCAT1) in Dr. Yuguang Shi's laboratory and his publications. Most of the antibodies used in this study were published/ validated by other research groups.

Eukaryotic cell lines

Policy information about cell lines and Sex and Gender in Research

Cell line source(s)	3T3-L1 cell is purchased from ATCC. Stromal-vascular cells were isolated from brown adipose tissue and inguinal adipose tissue of WT and Lcn2 KO C57Bl/6J mice and induced to differentiate into brown and inguinal adipocytes as previously described (Lin et al, 2020 Obesity; Deis et al 2018 Journal of Molecular Endocrinology).
Authentication	3T3-L1 cell is a well-established and commercially available (ATCC) cultured model of adipocyte differentiation. We follow a standard protocol from ATCC for the culture and differentiation of 3T3-L1 cells. For differentiated brown adipocytes, the lab people obtain training in isolation and culture of primary mouse stromal-vascular (SV) cells from the corresponding author who initially received training in these techniques during her PhD studies from Dr. Gary Hausman's laboratory at the University of Georgia and her postdoctoral work from Dr. Samuel Cushman's laboratory at the NIDDK. Briefly, primary SV cells are isolated from adipose tissue and used for adipocyte differentiation. Adipose tissue are removed from mice, minced, and digested with Krebs-Ringer bicarbonate HEPES buffer containing 2 mg/ml collagenase. After 1.5-h digestion, SV cells are separated from floating adipocytes through centrifugation at 1200 rpm for 5 min and washed with KRBH buffer twice. After the final wash, SV cells from WT and KO mice are plated at the same cell density on 6-well plates and cultured in DMEM containing 20% fetal bovine serum (Sigma) and 100 IU/ml penicillin/streptomycin (Invitrogen) until confluence. Cells are then treated with the differentiation cocktail consisting of DMEM, 10% fetal bovine serum (Sigma), 100 IU/ml penicillin/ streptomycin (Invitrogen), 115 g/ml methylisobutylxanthine (Sigma), 1 g/ml insulin (Sigma), 100 ng/ml dexamethasone (Sigma), 125 M indomethacin (Sigma), and 20uM L-3,3',5-Triiodothyronine. Three days later, the differentiation cocktail is replaced with DMEM containing 10% fetal bovine serum, 100 IU/ml penicillin/streptomycin, and 1g/ml insulin and cells are cultured for additional 6 days. On day 9 of differentiation, differentiated adipocytes are used for the experiments. For both 3T3-L1 adipocytes and brown adipocytes, the differentiation is authenticated by the morphology and adipocytes. Differentiated 3T3-L1 adipocytes and brown adipocytes exhibit typical morphology of adipocy tes with accumulation of lipid droplets.
Mycoplasma contamination	Cell lines were not tested for Mycoplasma contamination.
Commonly misidentified lines (See ICLAC register)	No commonly misidentified cell lines were used.

Animals and other research organisms

Policy information about studies involving animals; ARRIVE guidelines recommended for reporting animal research, and Sex and Gender in Research

Laboratory animals	8 week-old C57BL6/J mice were purchased from Jackson Laboratory. Lcn2-kockout (KO) mice were kindly provided by Dr. Alan Aderem, Institute for Systems Biology, Seattle, Washington, USA. Heterozygous mating scheme was used to generate WT and Lcn2 KO mice as previously described (Guo et al, 2010 Diabetes). WT and LcnN2 KO male mice were housed at 22°C, with free access to water, in a specific pathogen-free facility at the University of Minnesota (12-h light/ dark cycle, 60–70% humidity). Male WT and Lcn2 KO mice were used for experiments at 8-10 weeks of age. For more details, please see methods section or figure legends.
Wild animals	This study did not include wild animals.
Reporting on sex	All mice used in this study were male as described in the Methods.
Field-collected samples	No field-collected samples were used in the study
Ethics oversight	Animal studies were conducted with the approval of the University of Minnesota Animal Care and Use Committee and conformed to the National Institute of Health guidelines for laboratory animal care (IACUC 2102A38852).

Note that full information on the approval of the study protocol must also be provided in the manuscript.
